# Why non-technical skills matter in surgery. New paradigms for surgical leaders

**DOI:** 10.1007/s44250-022-00002-w

**Published:** 2022-09-12

**Authors:** Lorenzo Cobianchi, Francesca Dal Mas, Juan Manuel Verde, Alain Garcia-Vazquez, Jacopo Martellucci, Lee Swanstrom, Luca Ansaloni

**Affiliations:** 1 Department of Clinical, Diagnostic and Pediatric Sciences, University of Pavia, Via Alessandro Brambilla, 74, 27100 Pavia, Italy; 2 IRCCS Policlinico San Matteo Foundation, General Surgery, Pavia, Italy; 3 Department of Management, Ca’ Foscari University of Venice, Venice, Italy; 4 Institut Hospitalo-Universitaire (IHU), Strasbourg, France; 5 Careggi University Hospital, Florence, Italy

**Keywords:** COVID-19, Non-technical skills, Soft skills, Surgical leaders, Surgical education

## Abstract

The surgical literature is paying more and more attention to the topic of soft or non-technical skills (NTS), defined as those cognitive and social skills that characterize high-performing individuals and teams. NTS are essential in supporting surgeons in dealing with unexpected situations. During the COVID-19 pandemic, NTS have been considered crucial in defining situation awareness, enhancing decision making, communicating among groups and teams, and fostering leadership. With a “looking back and planning forward” approach, the current perspective aims at deepening the contribution of NTS for surgeons to deal with the unexpected challenges posed by the COVID crisis, surgical emergencies, the introduction of new technologies in clinical practice, to understand how such skills may help shape the surgical leaders of the future.

## Introduction

Among the multiple challenges derived from the COVID-19 pandemic to the healthcare and surgical systems, there was a clear need for certain qualities that had nothing to do with clinical or technical attitudes. Much knowledge has been shared about the clinical and organizational issues that surgical departments had to address. Now that a “new normal” has been reached [1], the surgical system is wrapping up the best practices and lessons learned during the crisis to redesign its protocols and procedures [2].

The COVID-related publications have exponentially increased the attention of the literature on the relevance of soft or non-technical skills (NTS), defined as “the cognitive and social skills that characterize high performing individuals and teams” [3] in supporting surgeons in dealing with unexpected situations [4, 5]. More in detail, NTS have been considered crucial in defining situation awareness, enhancing decision making, communicating among groups and teams, and fostering leadership [3]. A summary of some NTS for surgeons gathered from the surgical literature is provided in the following Table 1.

**Table 1:** A framework of soft or non-technical skills in surgical practice

Dimension	Category	Factors
Cognitive	Situation awareness	Acquiring information and data Understanding information and data Projecting and anticipating the future state-of-the-art and the risks
	Surgical and clinical decision making	Considering and evaluating the potential options Selecting and communicating the chosen option to the others Implementing and reviewing eventual decisions
Interpersonal	Leadership	Setting and maintaining standards Supporting and backing team members Coping with uncertain events, high pressure, fatigue, and stress
	Communication and teamwork	Sharing and translating information and knowledge Establishing a shared understanding Coordinating team members

Source: Modified from Yule S, Paterson-Brown S [33]

Surgical teams are often made up of a multidisciplinary group of people from various specialities, with a team leader coordinating their activities [4, 6]. As a result, it appears that recognizing the different NTS required to work in such a setting is also essential. Although the ability to put certain decisions into action is frequently perceived through the prism of an individual’s internal cognitive mechanism, the ability to seamlessly coordinate all team members to accomplish the desired goal necessitates the ability to put certain decisions into action [7]. Knowledge translation and knowledge sharing processes appear to be critical in this situation, allowing team members to bridge their gaps and collaborate effectively, enhancing multidisciplinary and diverse teams to reach their full potential and improve patients’ outcomes [8].

With a “looking back and planning forward” approach, aiming at understanding what we have learned from the pandemic and what may be relevant for planning the future of surgery, we want to deepen the contribution of NTS for surgeons to deal with the challenges of the COVID crisis, surgical emergencies, new technology introduction in clinical practice, to understand how such skills may help shape the surgical leaders of the future. The single skills were not considered individually but in specific complex situations typical in today’s practice. The analysis of these situations can provide readers with an idea of how NTS are not independent entities but are part of a global personal attitude. Employing a narrative review of the literature, our paper examines a potential framework of analysis of NTS applied to some relevant COVID-related dimensions, followed by a summary of NTS-related topics like NTS in clinical practice, education, and how to assess and evaluate them. In concluding our work, we summarize some future perspectives related to NTS for the surgical leaders of the future.

## NTS in the COVID experience

**Fig. 1:**
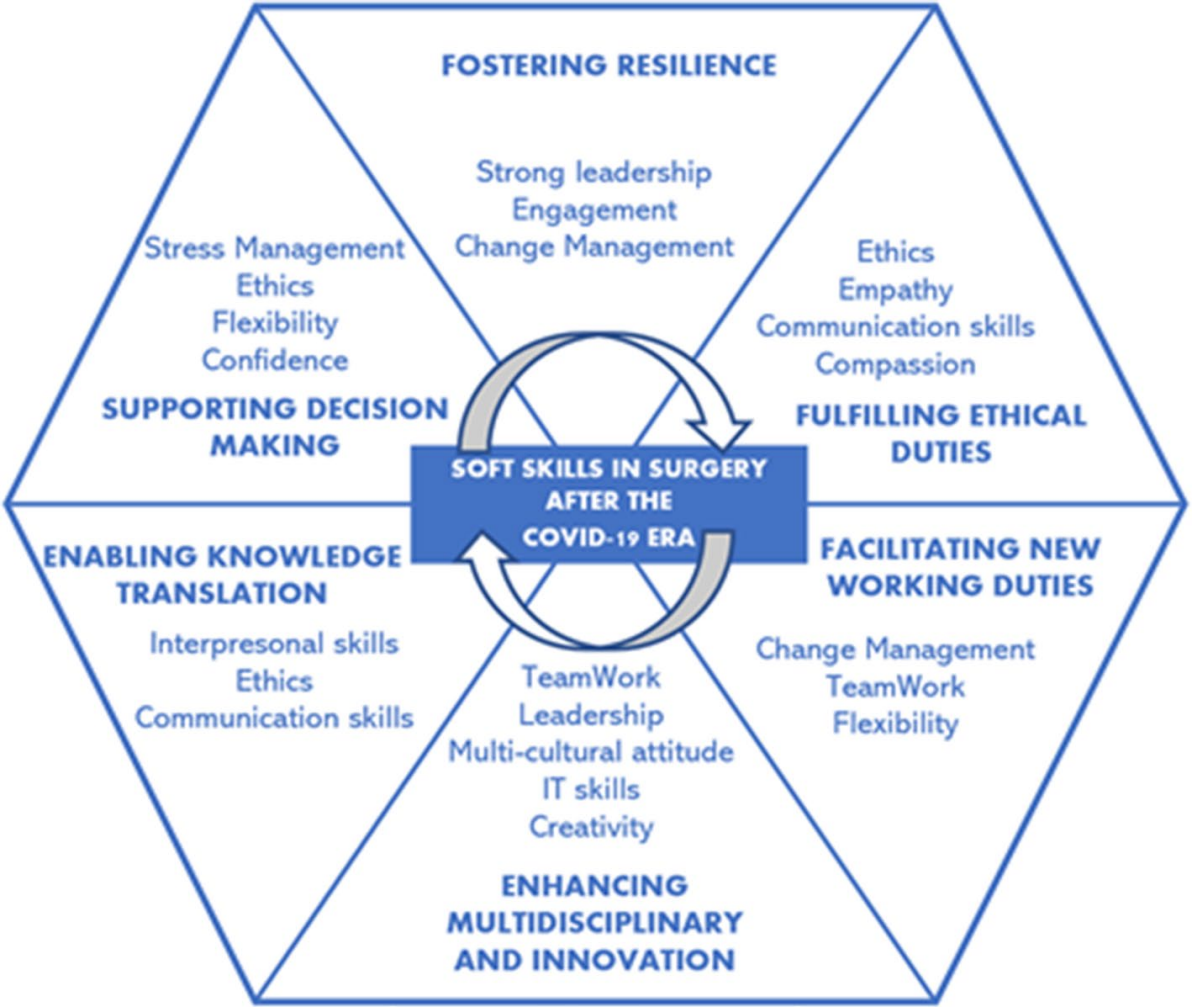
NTS/Soft skills in surgery after the COVID-19 era. Source: Authors’ elaboration

Starting from the recent COVID experience and a narrative review of the COVID literature, our roadmap analyzes the contribution of NTS to the following aspects: resilience; ethical duties; new working duties; multidisciplinarity and innovation; knowledge translation; decision making and perception awareness. NTS in surgery according to such elements are summarized in the following Fig. 1.

### Resilience

According to the literature, during the Emergency and Transition phases of the COVID era, healthcare institutions aimed at resilience [1], trying to cope with the presence of the virus while doing their best to ensure urgent services, including surgical procedures [9–11]. Resilience is usually associated with an organization’s survival in the face of unexpected change [11, 12]. The literature linked organizational resilience to several attributes, like strong leadership, engagement, and change management, which contribute to the process of building resilience [12].

In the case of surgeons, resilience may be separated into two different levels:

1. self-resilience, required to cope with stress aiming to decrease its impact. In this regard, new skills were needed, out of the usual surgical environment, treating different patients with a totally new and unknown disease, which did require, for instance, even finding “homemade” technical solutions to be protected from it [13–16];
2. team-resilience, in which surgeons can contribute with valuable skills to enhance team-playing (networking, team working, communication, leadership, among others) [3–5, 17].

### Ethical duties

During the COVID pandemic, the ethical dimension involved the whole system, which shifted from patient-centred ethics to public health ethics [9]. Surgical ethical duties increased during the pandemic, including the need to decide which surgical procedures to carry on [18] and further implications related to consent for hospitalization [19]. Ethical behaviour is considered itself a soft skill, which should be coupled with empathy, compassion, and communication skills to deal with patients [20, 21].

### New working duties

Several surgical staff had to move to COVID wards and intensive care units (ICUs) to help in the care of COVID patients [1]. Surgeons had to change their routines, leaving most of their expertise behind and quickly gaining new technical skills by undergoing on-the-job training or being coordinated by colleagues with different specialities, like anesthesiologists and palliative care specialists [22]. Surgeons had to be included in teams they were not familiar with, with colleagues they did not know, taking charge of tasks and duties far from their daily practice, with no or little time to learn. Change management, the attitude towards teamwork, and flexibility in the workplace facilitated the change in the role to ensure the outcome. Communication skills were needed while following up with patients that required the postponement of oncological surgery, explaining to them the delay of the operation and the eventual consequences [23].

### Multidisciplinarity and innovation

Multidisciplinarity and innovation proved to be vital topics in overcoming the crisis, even in surgery [8, 11, 13, 15]. Successful examples come from the COBRA multidisciplinary experience of the Massachusetts General Hospital in Boston, MA [14] and the Italian experience of Venturi/Charlotte valves that allowed a snorkelling mask to be converted into a ventilation device for COVID patients [15].

Working in multidisciplinary teams and dealing with innovation can be enhanced by the ability to teamwork, the attitude to leadership, an open mind leading to a multicultural approach, creativity, and proficiency in Information & Communication Technologies (ICT).

### Knowledge translation

Knowledge translation is the ability to transfer and share knowledge despite the differences in the characteristics of the stakeholders involved [22, 24]. The COVID crisis pushed surgeons to deal with the translation of clinical and organizational knowledge within the surgical community, the need to work in multidisciplinary medical teams, and the necessity to manage a different relationship with patients whose surgical procedures had to be postponed [22]. The literature highlighted the influential role of some NTS in acting as knowledge translation enablers and facilitators [21, 22, 25]. Surgeons with attitudes towards ethics, communication, and other interpersonal skills proved to manage the knowledge translation processes more efficiently [2].

The literature has stressed that during surgical training, the most valued NTS are character (approachability, patience, enthusiasm, supportiveness), communication skills and time availability and operative control (balance between trainee supervision and independence) [26]. However, due to the lack of onsite training, the challenge remains on how to continue transferring the abovementioned soft skills by setting up remote and online courses [27].

### Decision making

Surgical decision-making can be defined as “Skills for diagnosing a situation and reaching a judgement in order to choose an appropriate course of action” [3]. Decision-making happens every day in surgical practices, and it looks particularly challenging in some settings like trauma and emergency surgery [5, 7]. Still, the COVID pandemic shifted the situation needing judgement by, for instance, deciding which surgical procedures to postpone or to be considered eligible [23]. While surgeons could mainly count on official guidelines and open-source clinical publications [1], unknown COVID-related events occurred [17]. Stress and change management skills, flexibility, confidence, the ability to cope with fatigue, and ethics in the workplace could support surgeons in evaluating the alternatives, limiting their biases, and forcing them towards shared-decision making with other professionals involved. While time-constrained and “non-delayable” decisions are part of a surgeon’s everyday job, dedicated training may enhance such aspects.

## NTS in clinical practice

A skilful operation is 75% decision making and 25% dexterity [28, 29], and in about half of the errors in surgical operations, there is a communication problem that contributes to the adverse surgical result [30]. This is why, in line with the old masters’ considerations, we talk increasingly more about attitude rather than technical ability when considering a surgeon’s needed skill set.

Communication is the cornerstone of teamwork [31], and a strong relationship has been noted between postoperative complications and poor team communication [32].

Team communication aims to share the same perception of a situation, considering available information, clinical options, risks, operative steps and potential outcomes. However, the presence of several communication limitations and barriers was reported [33]. These obstacles or barriers can be divided in “internal”, if related to personal limitations, such as cultural, motivational, personal experiences and training, hierarchical conflicts, stress, fatigue, or mood, or “external”, if related to environmental issues, such as noise, the distance between the staff, low voices, absence of visual contact, hearing limitations. All in all, adequate communication should be considered a goal of the whole group, not just of the individual.

The team analysis of errors is recognized to be essential but often perceived as challenging to discuss together and not always well managed [34]. Moreover, errors analysis is more critical when stress and fatigue on performance are considered.

It is a well-known and daily experience that fatigue and stress are two elements that can easily affect NTS, regardless of technical factors. Sleep deprivation, even minimal, can have effects on cognitive abilities [35] comparable to alcohol consumption [36]. Despite that, the real impact of fatigue on surgical performance and NTS remains underestimated, considering that up to 70% of surgeons, when questioned, claim that fatigue does not affect the technical gesture. Moreover, it has been suggested that in laparoscopic surgery, complications (such as bleeding) or time pressure on daily activity may be considered to be major sources of stress for a surgeon [37] even if a surgeon's exposure to stress is probably chronic, often related to constant anxiety of a possible complication [38].

In emergency and trauma contexts, NTS have proved to be essential in facilitating communication among team members, especially when there is little time to decide or when the patient’s life is in danger [5, 6, 33, 39]. According to a recent investigation [4, 40], trauma and emergency surgeons underline many difficulties in efficiently conducting their work within their assigned teams. The main issues concern a lack of trust with colleagues and authoritarian and generally demanding relations with their team leaders. Also, multidisciplinarity, which appears as a pillar in emergency and trauma surgery and other medical disciplines (like oncology), is seen more as a liability than an asset, with surgeons struggling to communicate with each other. Therefore, enhancing those NTS like leadership, teamwork, communication, ethics, and empathy look essential to foster the performance of trauma and emergency teams.

Further issues that may affect NTS and team communication usually include differing perceptions of teamwork among team members and the reluctance of senior staff to accept input from junior members. The concept of senior vs junior unidirectional hierarchy is deeply rooted in hospital culture, and particularly in surgery [41, 42]. Like in aeronautic experience, the “calling into question” and active participation should be encouraged. Interesting enough, these challenges were underlined during the first waves of the COVID pandemic, when several surgeons were temporarily moved to COVID wards and had to work under the leadership of other clinical professionals, including nurses and palliative care physicians [22].

Today’s surgical practice is seeing the importance of new surgical techniques using the latest Industry 4.0 technologies [43] like robotics, virtual and augmented reality, artificial intelligence (AI) and machine learning [44, 45]. Engaging in such new techniques may not seem easy, especially when there is a misalignment between surgical education and the new skills required to use such advanced tools. For instance, surgeons’ guesswork should be replaced by the ability to interpret surgical data reported by the new instruments [43, 46]. Therefore, NTS, like the ability to learn, deal with ICT, and change management attitudes, look fundamental to coping with the transition.

## NTS education

Despite the recognized value of NTS in surgical practice, these are rarely considered during surgical or medical education. Young surgeons are supposed to learn NTS elements simply by observing senior surgeons and their behaviour without any specific training. However, it has been shown that focusing on NTS training can potentially have a relevant impact on adverse events number, resulting in reduced patient morbidity and mortality and improved outcomes [47].

Two different kinds of training for NTS were reported: a simulation-based model, finalized by structured discussions and event analysis, or a didactic lecture model, implemented by interactive instruction support. The highest level of training impact was found for the simulation model, showing a significant positive effect on NTS, in particular communication and coordination, as reported in the analysis of two systematic reviews [48, 49].

Integrating NTS and technical skills training, ideally completed with follow-up in the clinical setting, in a complete simulation program may maximize the learning effect. New exciting tools may come from the Metaverse and simulation tools like the one of OSSO VR [50]. In this simulated OR model, trainees could play different roles, learning to better understand the various interprofessional issues. Moreover, training various NTS in a simulated daily practice and analyzing behaviour and mistakes with the help of senior surgeons may help to have a full comprehension of all the complex dynamics of a surgical setting, really understanding how NTS may influence surgical performance.

This kind of simulation-based training model has proven to be realistic and reliable for training teamwork and other NTS among surgical residents [51]. The possibility to consider NTS as something helpful to improve besides manual and technical abilities is essential. It needs to be considered for routine surgical resident training to enhance performance and safety in surgery. Innovative ways as gaming and gamification could also support the enhancement of such skills [52].

## NTS evaluation

Starting from the reviews published on NTS (especially in the OR) and from aeronautic experiences, based on the classification of skills and non-technical behaviours that may affect surgical performance, some systems for NTS evaluation in surgery have been realized and summarized in Table 2.

**Table 2:** NTS evaluation methods in surgery

System	Categories	Main features
NOTSS Non technical skills for surgeons	Situation awareness Decision making Communication Teamwork & leadership	Created with an interview of 27 consultant surgeons in different specialities Requires appropriate training [5, 53–55]
NOTECHS Non-TECHnical Skills	Leadership & management Teamwork & cooperation Problem-solving & decision making Situational awareness	Adjusted from experience in the aviation sector with crew resource management training [56] Modified adding one different category, “communication and interaction” based on OR different professionals figures involvement [51]
OTAS Observational teamwork assessment for surgery	Teamwork-related task checklist: [Patient tasks, equipment/provisions tasks, communication tasks] Teamwork‐related behaviours: [Communication, cooperation, coordination, leadership, monitoring]	15 items with a 7-point scale Developed from the need to have a complete evaluation of all the factors involved in patient outcomes[57]
OSANTS Objective structured assessment of non-technical skills	Situation awareness Decision making Teamwork Communication Leading and directing Professionalism Managing & coordinating	Developed and applied for residents and for the training of NTS [58] 5-point rating scale range Reliable and effective even in research and education Few studies regarding the reliability and validity of this assessment compared to NOTSS and NOTECH

Systems like NOTECHS (Non-TECHnocal Skills) or OTAS (Observational Teamwork Assessment for Surgery) provide a whole team assessment, while other systems like NOTSS (NOn Technical Skills for Surgeons), OSANTS (Objective Structured Assessment of Non-technical Skills) or ANTS (focused on anesthesiologists) are more focused on sub-team performances.

## Conclusion and future perspectives

The analysis highlights the relevance of soft or NTS for surgeons in managing the COVID crisis. Thus, such skills represent key assets, besides pure technical knowledge, for the next surgical leaders. Still, there are some open questions that should be addressed.

The literature has struggled to answer the need to understand how much personal traits and habits can influence the possession or not of certain soft skills [59], and thus how much NTS training can overcome human nature. All in all, the main question is, how much can soft skills be learned as technical skills are? One solution may be to acknowledge the lack of specific skills, which are highly needed in some contexts (like public speaking ability in academia), and thus receive dedicated training. Modern training techniques have proved to be successful in other settings, like in the case of entrepreneurship, where professional poker players teach students how to play and gamble to be able to cope with entrepreneurial risk. Pioneering procedures include gaming and gamification techniques with the support of the latest technologies [50, 52] and sports.

We then argue that dedicated competencies mapping and soft skills training should be put in place to provide the next surgical leaders with all the needed expertise to face the challenges of the new surgical system. According to their specific role, surgeons should acknowledge which soft skills may be more relevant for them, understanding how these are already embedded in their traits and how to foster them through dedicated learning paths. Such an assessment can be self-made or done with the support of their home institution.

## Data Availability

None.
